# The predictive role of platelet-to-lymphocyte ratio and systemic immune-inflammation index in young and middle-aged patients with tibial plateau fractures

**DOI:** 10.3389/fsurg.2025.1654222

**Published:** 2025-09-16

**Authors:** Shaowei Zhou, Xuemei Yang, Fang Hu, Xiaomeng Dong, Qingcheng Song, Shuhong Yang, Yingze Zhang

**Affiliations:** ^1^ Department of 2nd Operating Room, Hebei Medical University Third Hospital, Shijiazhuang, Hebei, China; ^2^ Shijiazhuang Obstetrics and Gynecology Hospital, Shijiazhuang, Hebei, China; ^3^ Department of Orthopaedic Surgery, Hebei Medical University Third Hospital, Shijiazhuang, Hebei, China

**Keywords:** tibial plateau fractures, platelet-to-lymphocyte ratio, systemic immune-inflammation index, fracture severity, imaging classification

## Abstract

**Background:**

There is suggestive evidence that the platelet-to-lymphocyte ratio (PLR) and systemic immune-inflammation index (SII) are related to the severity of fracture. The purpose of this study was to investigate the role of PLR and SII in predicting fracture severity in young and middle-aged patients with tibial plateau fractures (TPFs).

**Methods:**

A retrospective cohort study involving 229 patients with isolated TPFs was conducted between January 2015 and December 2019. Medical records of hospitalized patients were extracted from the electronic case system. Three experienced orthopedic surgeons classified the imaging data according to the Schatzker classification. All the patients were divided into two groups: group1 consisted of fractures of mild to moderate severity (Schatzker types I-IV), and group2 consisted of fractures of severe severity (Schatzker types V-VI). Platelet, neutrophil, and lymphocyte values at admission were obtained. The PLR = platelet/lymphocyte counts and the SII = platelet × neutrophil/lymphocyte counts were noted. Patients in groups 1 and 2 were statistically compared in terms of PLR and SII value on hospital admission.

**Results:**

There were significant differences in the blood PLR, SII, Na^+^ and K^+^ levels, and neutrophil count between the two groups. According to the receiver operating characteristic (ROC) curve, the cut-off of PLR and SII were 157.9 and 923.9, respectively. Our results showed that high PLR and SII were remarkably associated with the severity of TPFs. The sensitivity was 60% and the specificity was 86.9% when using the PLR ≥ 157.9 to predict the severity of the TPFs whereas the sensitivity was 63.3% and the specificity was 74.4% to predict the severity of TPFs at SII ≥ 923.9. In the multivariate analyses, the high preoperative PLR and SII were identified as independent predictors of severe TPFs.

**Conclusions:**

The PLR and SII are simple and economical biomarkers that require only routine blood tests with low associated costs. They can be calculated directly from platelet, neutrophil, and lymphocyte counts in standard blood routine reports, making them readily accessible and cost-effective tools to predict the severity of tibial plateau fractures.

## 1 Background

The tibial plateau serves as one of the primary load-bearing structures in the human body, capable of withstanding mechanical forces equivalent to five times body weight during dynamic activities (1). This anatomical region is inherently susceptible to external traumatic forces, which can lead to soft-tissue injuries compromising knee joint mobility and biomechanical stability (2–4). Tibial plateau fractures (TPFs) represent a subset of complex intraarticular fractures, typically resulting from high-energy trauma, and pose significant challenges to trauma surgeons due to their intricate fracture patterns and the concomitant risks of neurovascular injury, delayed union, or non-union (5). Epidemiological studies have highlighted that TPFs predominantly affect the young and middle-aged demographic, underscoring the clinical urgency to develop optimized therapeutic strategies for this patient population (6, 7).

Radiological classification systems of fractures, designed to group fractures according to injury mechanisms and fracture patterns, have been utilized to assess the severity of fracture injury (8). Among these, the Schatzker classification, based on two-dimensional radiographic interpretation, remains the gold standard for tibial plateau fractures (TPFs), systematically categorizing them into six distinct subtypes (I-VI) (2). Clinically, an increase in Schatzker classification grade correlates with progressively severe traumatic forces, heightened knee joint instability, and worse long-term prognoses, reflecting the escalating complexity of osseous and soft-tissue involvement (9).

Recent investigations have underscored the utility of specific hematological markers as potential biomarkers for gauging tissue damage severity. The platelet-to-lymphocyte ratio (PLR), defined as the absolute platelet count normalized by absolute lymphocyte count, has emerged as a robust predictor in diagnosing and prognostication across diverse clinical contexts—including hip fractures, inflammatory disorders, oncological diseases, systemic lupus erythematosus, and cardiovascular pathologies (10–13). Mechanistically, traumatic immune responses rely on a delicate equilibrium between pro-inflammatory and anti-inflammatory signaling networks (14). Severe trauma disrupts this homeostasis, triggering a systemic immune dysregulation characterized by peripheral neutrophilia and lymphopenia. This immunological paradigm rationalizes the use of the systemic immune-inflammation index (SII) as a surrogate marker for quantifying trauma severity, reflecting both the magnitude of tissue injury and the ensuing immune cascade.

However, the association between PLR, SII levels, and the severity of tibial plateau fractures (TPFs) remains underexplored in the literature, with limited mechanistic insights into their predictive utility. Therefore, this study aims to systematically evaluate the prognostic value of PLR and SII in young and middle-aged TPF patients, with a focus on deciphering their clinical relevance in guiding fracture management. By integrating hematological markers with radiological classifications (e.g., Schatzker types), this investigation seeks to establish a novel biomarker-based framework that may optimize risk stratification and improve patient outcomes in this vulnerable demographic.

## 2 Methods

### 2.1 Study design and patients

This retrospective study was conducted in the level I electronic case system, spanning five years (between January 2015 and December 2019). The enrolled participants were young and middle-aged adults between 18 and 60 years, the diagnosis was unilateral isolated closed TPF, and blood test and imaging records were complete. The exclusion criteria were: (a) participants with cardiovascular disease, malignancies, autoimmune disease, multiple traumas, open wounds, postoperative infections, severe neurovascular injury, and systemic inflammatory or infectious diseases, (b) participants whose time from injury to admission were >48 h. A total of 229 patients with isolated TPFs were included in the study.

### 2.2 Imaging data and patient grouping

All images of the patients were taken from the hospital's Picture Archiving and Communication Systems (PACS). Patients who met the inclusion criteria had their images read independently by three experienced orthopedic surgeons. The surgeons worked independently and did not communicate with each other. The imaging data of all patients with tibial plateau fractures were classified according to the Schatzker classification system. Types I–III are fractures of the lateral tibial plateau, whereas Type IV is the isolated fracture of the medial column of the tibial plateau. Types I–IV are simple fractures that are often associated with minor injuries, whereas Type V–VI are complex fractures and are often associated with more violent injuries and a significant compromise of the soft tissue envelope (2). Therefore, the patients were divided into two groups: Group 1 was mild to moderate TPFs, representing patients of Schatzker type I–IV fractures, whereas group 2 was severe TPFs, representing patients of Schatzker type V–VI fractures.

### 2.2 Data collection

Patient characteristics extracted from the electronic medical records included: Demographic characteristics, lifestyle risk factors, comorbid diseases, laboratory data such as white blood cell count (×10^9^/L, reference range 3.5–9.5), platelet counts (×10^9^/L, reference range 100–300), lymphocyte counts (×10^9^/L, reference range 1.1–3.2), albumin (g/L, reference range 35.0–55.0), creatinine (μmol/L, reference range 53.0–106.0) and C-reaction protein (mg/L, reference range 0.5–8) (Table 1). The patient's venous blood was drawn by a trained nurse on admission. The PLR = platelet/lymphocyte counts, SII = platelet × neutrophil/lymphocyte counts, and the Schatzker classification was used to assess the severity of TPFs.

**Table 1:** Clinicopathological characteristics of patients with TPFs.

Variable	Group1 (*n* = 180)	Group2 (*n* = 49)	*P*-value
Gender (male)	105 (58.3%)	29 (59.2%)	1.000
Age (years) mean ± SD	39.65 ± 11.54	45.55 ± 9.46	
18–45	99 (55%)	23 (47%)	0.336
46–60	81 (45%)	26 (53%)	
Smoking	31 (17.2%)	7 (14.3%)	0.674
Alcoholism	25 (13.9%)	9 (18.3%)	0.496
Hypertension	31 (17.2%)	11 (22.4%)	0.409
Diabetes	16 (8.9%)	1 (2%)	0.131
Time from injury to admission (h)			0.807
0–24	157 (87.2%)	44 (89.8%)	
25–48	23 (12.8%)	5 (10.2%)	
Neutrophil count (>6.30 × 10^9^/L)	76 (42.2%)	27 (55.1%)	0.144
Lymphocyte count (<1.10 × 10^9^/L)	21 (11.7%)	7 (14.3%)	0.626
PLT (10^9/L)			1.000
125–350 reference	168 (93.3%)	45 (91.8%)	
<125	9 (5%)	3 (6.1%)	
>350	3 (1.7%)	1 (2.1%)	
ALT (>50 U/L)	11 (6.1%)	1 (2.1%)	0.317
AST (>40 U/L)	4 (2.2%)	0 (0%)	0.580
WBC (> 10 × 10^9^/L)	21 (11.7%)	5 (10.2%)	0.810
PLR mean ± SD	134.14 ± 54.45	204.93 ± 53.79	<0.001
SII mean ± SD	836.06 ± 500.96	1,011.72 ± 495.16	0.012
ASA			0.326
1	148 (82.2%)	40 (81.6%)	
2	28 (15.6%)	6 (12.2%)	
3	4 (2.2%)	3 (6.2%)	
TP (<60 g/L)	108 (60%)	34 (69.4%)	0.250
ALB (<35 g/L)	97 (53.9%)	27 (55.1%)	1.000
A/G			0.508
1.2–2.4 reference	167 (92.8%）	44 (89.8%)	
<1.2	1 (0.56%)	0 (0%)	
>2.4	12 (6.64%)	5 (10.2%)	
HCRP (>8 mg/L)	141 (78.3%）	39 (79.6%)	1.000
UA (>428 μmol/L)	34 (18.9%)	6 (12.2%)	0.301
CREA (>73 μmol/L)	35 (19.4%)	9 (18.4%)	1.000
Na^+^ (mmol/L)			0.016
137–147 reference	162 (90%)	37 (75.5%)	
<137	17 (9.4%)	12 (24.5%)	
>147	1 (0.6%)	0 (0%)	
K^+^ (mmol/L)			0.001
3.5–5.3 reference	149 (82.8%)	49 (100%)	
<3.5	30 (16.7%)	0 (0%)	
>5.3	1 (0.5%)	0 (0%)	
Cl^−^ (mmol/L)			0.319
99–110 reference	166 (92.2%)	42 (85.7%)	
<99	6 (3.3%)	3 (6.1%)	
>110	8 (4.5%)	4 (8.2%)	

### 2.3 Statistical analysis

**Figure 1:**
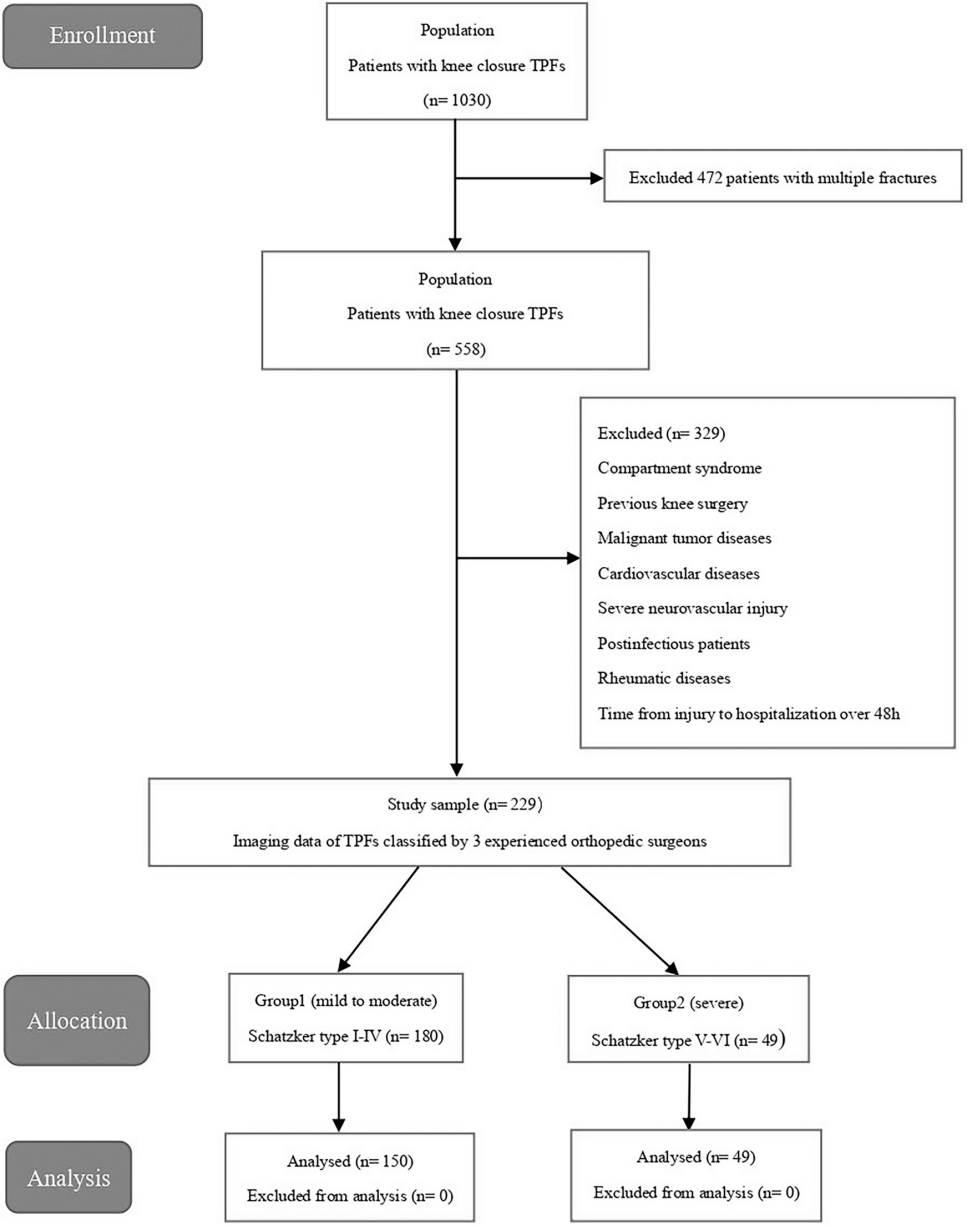
Flow diagram of patients included in this study.

All statistical analyses and graphics in this study were analyzed using SPSS21.0 (IBM Corp, Armonk, NY, USA). Continuous data were expressed as mean ± standard deviation (SD) and categorical variables were expressed as absolute values and percentages. Student's *t*-test was used for continuous variables conforming to the normal distribution, and the chi-square test was used for categorical variables to compare the differences between the two groups. Univariate analysis was used to screen the risk factors of TPFs. Factors with *P* < 0.2 in univariate analysis were further analyzed using multivariate logistic analysis. Odds ratio (OR) and 95% confidence interval (CI) were used to measure the strength of association between risk factors and TPFs. Moreover, the ROC curve was used to calculate the cutoff points of blood PLR and SII. In the multiple logistic regression analysis, *P* <0.05 was considered to be significantly associated with TPFs and was included in the model. For all statistical tests, *P* < 0.05 was considered statistically significant (Figure 1).

## 3 Results

Over the five years (January 2015–December 2019), 229 patients were identified with TPFs eligible for inclusion criteria comprising 134 (58.5%) men and 95 (41.5%) women. The average age of the patients was 43.20 (SD 10.46). The patients were divided into two groups according to the Schatzker classification: Group 1 (*n* = 180) included patients with mild to moderate TPFs (Schatzker types I–IV), and Group 2 (*n* = 49) included patients with severe injuries (Schatzker types V–VI). Demographic and clinical characteristics are shown in Table 1.

**Figure 2:**
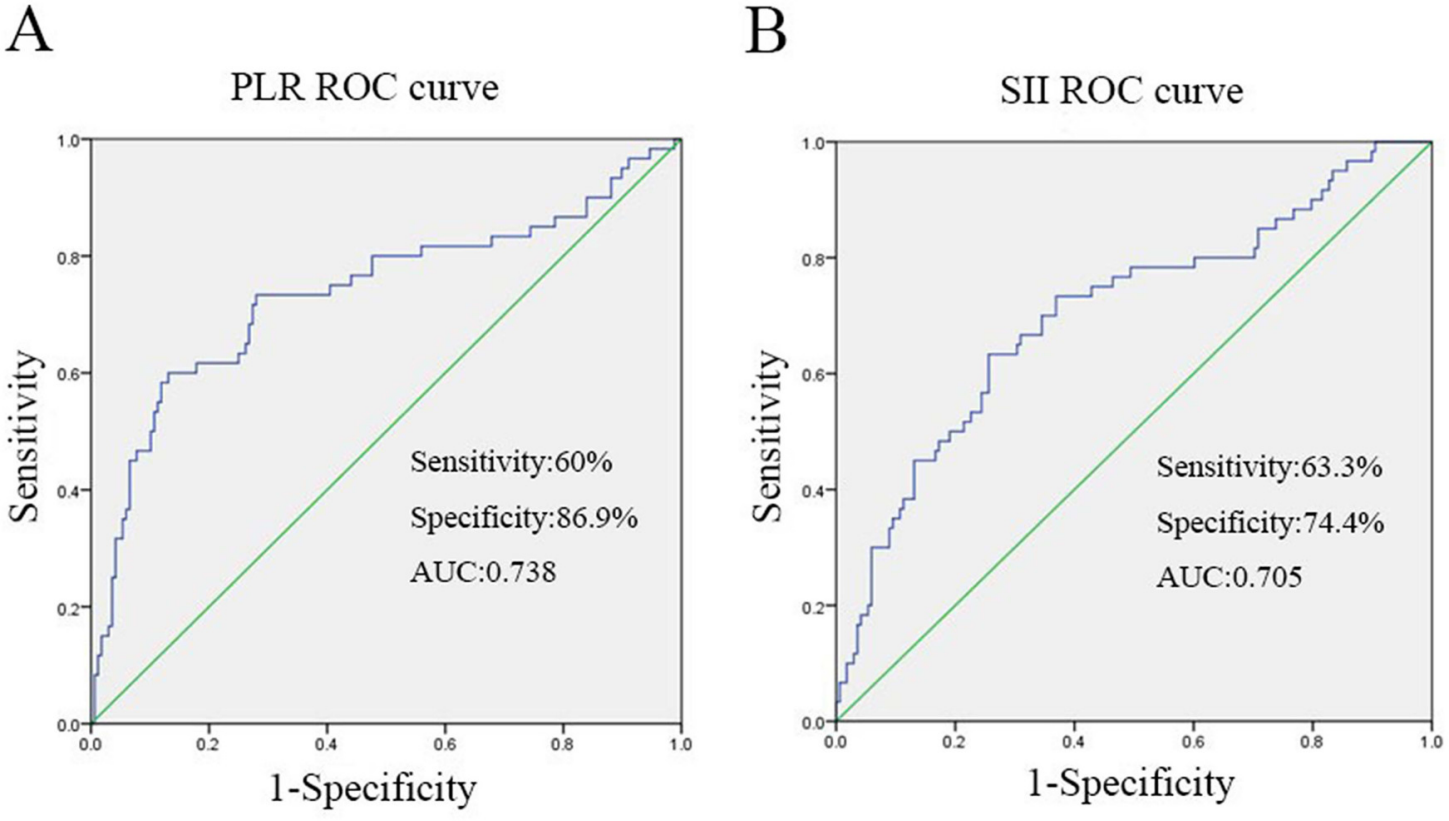
The figure was produced by SPSS21.0 (IBM Corp, Armonk, NY, USA). **(A)** ROC curve analysis when the PLR cut-off point was 157.9, the sensitivity and specificity of PLR for predicting severe TPFs on admission were 60% and 86.9%, respectively, the area under curve was 0.738. **(B)** When the SII cutoff point was 923.9, the sensitivity and specificity of SII to predict severe TPFs at admission were 63.3% and 74.4%, respectively, the area under curve was 0.705.

**Figure 3:**
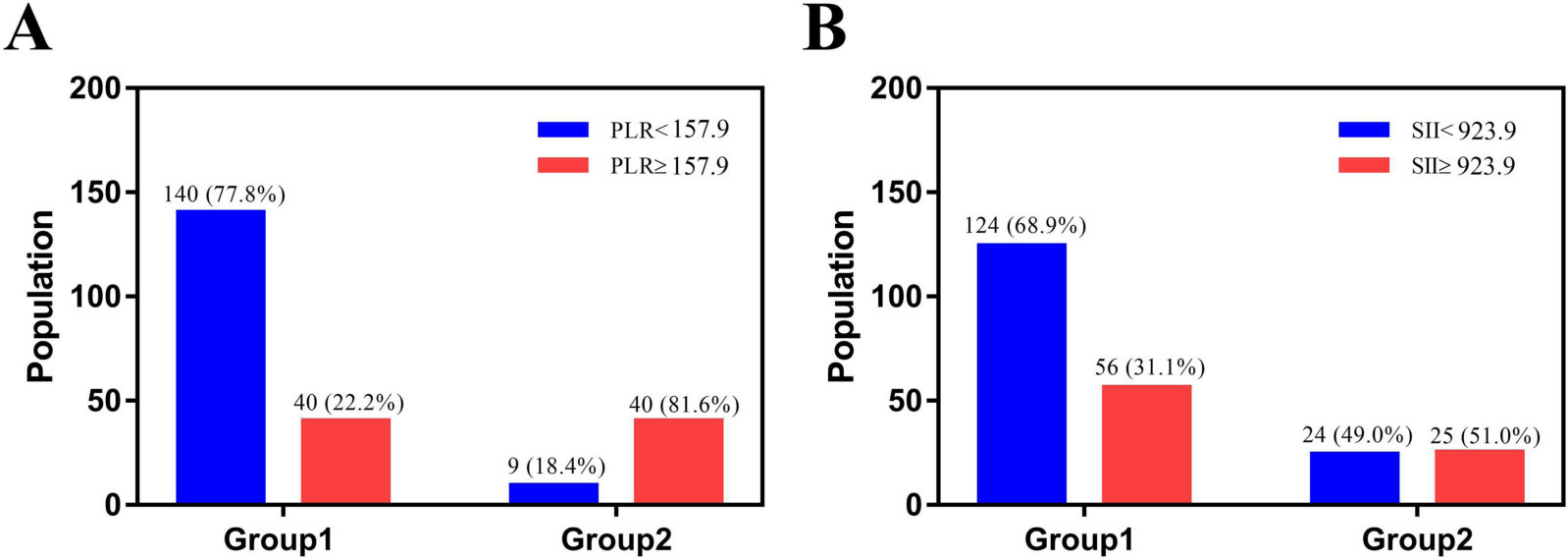
The image was produced by GraphPad Prism 5.0. **(A)** The number and percent of patients with PLR ≥157.9 were 40 (22.2%) in group 1 and 40 (81.6%) in group 2. **(B)** The number and percent of patients with SII ≥ 923.9 were 56 (31.1%) in group 1 and 25 (51%) in group 2.

The ROC curve was employed to determine the optimal cutoff values of PLR and SII for predicting the severity of TPFs. For Group 2 (severe fractures), the area under the curve (AUC) for PLR was 0.738 [95% confidence interval (CI): 0.654–0.821, Figure 2A], while the AUC for SII was 0.705 (95% CI: 0.624–0.785, Figure 2B). Univariate analysis revealed significant differences between severe and mild-to-moderate TPF patients in the following parameters: PLR ≥ 157.9 (*P* = 0.046), SII ≥ 923.9 (*P* = 0.011), serum sodium (>147 mmol/L, *P* = 0.024), and serum potassium (<3.5 mmol/L, *P* = 0.241). Notably, the proportions of patients with PLR ≥ 157.9 and SII ≥ 923.9 in Group 2 were 81.6% and 51%, respectively, both of which were significantly higher than that of PLR ≥157.9 (22.2%) and SII ≥ 923.9 (31.1%) in group 1 (Figure 3).

Tibial plateau fracture (TPF) represents a prevalent intraarticular injury, predominantly affecting young adults and males, often resulting from high-energy traumatic events, such as bicycle collisions or motor vehicle accidents, which can lead to serious knee movement problems, therefore, age and gender were included in the multiple regression analysis. Multiple logistic regression modeling revealed that PLR ≥ 157.9 and SII ≥ 923.9 were independent risk factors for severe TPFs, after adjusting for confounding variables (Table 2).

**Table 2:** Multivariate analyses for TPFs.

Variable	Multivariate analysis		*P*-value
	OR	95% CI	
PLR ≥ 157.9	2.205	1.014–4.795	0.046
SII ≥ 923.9	2.307	1.213–4.387	0.011
Na + (>147 mmol/L)	0.340	0.134–0.866	0.024
Diabetes	0.084	0.01–0.712	0.023
Neutrophil count (>6.30 × 10^9^/L)	1.147	0.525–2.505	0.731
K^+^ (<3.5 mmol/L)	1.916	0.646–5.681	0.241

## 4 Discussion

To the best of our knowledge, this study represents a systematic exploration of the association between PLR, SII levels, and TPF severity. Our findings demonstrate that patients with severe TPFs (Schatzker types V–VI) exhibit significantly elevated PLR and SII values compared to those with mild-to-moderate fractures (types I–IV). These results highlight PLR and SII as cost-effective, readily measurable laboratory markers that not only reflect radiological fracture severity (as defined by Schatzker classification) in young and middle-aged populations but also serve as robust predictors of TPF severity. By integrating these hematological indices with traditional radiological assessments, clinicians may enhance risk stratification for TPF patients, potentially optimizing treatment strategies and improving clinical outcomes.

The tibial plateau fracture is a prevalent intraarticular injury characterized by fracture lines involving the proximal tibia, accounting for 1%–2% of all skeletal fractures. Such injuries frequently lead to knee joint mobility disorders and instability. The Schatzker classification, a widely adopted system based on two-dimensional radiographic imaging, effectively reflects TPF severity by delineating six distinct fracture subtypes. This classification framework has been validated and universally recognized as a practical criterion for clinical decision-making. However, its reliance on detailed morphological assessment introduces inter- and intra-observer variability, with disagreement rates increasing as classification complexity rises (2). This subjectivity highlights the dependency of the Schatzker system on clinicians' experience and familiarity with its criteria, necessitating adjunctive objective markers for improved diagnostic consistency.

In recent years, the platelet-to-lymphocyte ratio (PLR) and systemic immune-inflammation index (SII) have emerged as prominent immunoinflammatory biomarkers, owing to their accessibility and cost-effectiveness in routine clinical practice. Extensive research has demonstrated the prognostic significance of PLR in predicting disease severity and mortality across diverse medical fields, including hip fractures, inflammatory disorders, malignancies, systemic lupus erythematosus, and cardiovascular diseases (10–12, 15). Moreover, the SII has been associated with the prognosis of malignancies, post-traumatic thrombosis, and fractures following osteoporosis (16–18). These findings underscore the potential of PLR and SII as versatile, readily available indicators capable of reflecting immune dysregulation and disease progression, positioning them as valuable tools for risk stratification and clinical decision-making. Platelets are not merely essential for hemostasis; they also act as crucial immunomodulatory agents. These cells contain a diverse array of soluble and cell-associated immunomodulatory molecules, which are rapidly released upon tissue injury (19). Upon activation, platelets can bind to leukocytes and vascular endothelial cells, initiating a cascade of cellular responses that significantly influence immune defense and inflammatory processes. This interaction can either amplify the inflammatory response, promoting the recruitment and activation of immune cells, or trigger cellular apoptosis, depending on the injury context and the balance of signaling pathways involved. As such, platelets serve as a pivotal link between hemostasis and the immune system, highlighting their multifaceted role in the body's response to damage (20, 21). Numerous investigations have demonstrated that physiological stressors—including tissue injury, severe trauma, and major surgical procedures—elicit a robust leukocytosis, typified by a neutrophilic shift in the peripheral blood (6). This immunological response, often referred to as the acute phase reaction, reflects the body's attempt to combat infection and facilitate tissue repair. For instance, a seminal study by Morell et al. established a direct correlation between white blood cell counts and Injury Severity Score (ISS), highlighting the quantitative relationship between systemic inflammation and trauma severity (22). This finding underscores the utility of leukocyte parameters as surrogate markers for evaluating the magnitude of tissue injury, providing clinicians with objective indices to complement subjective clinical assessments. Moreover, Neutrophils can promote tissue repair after injury by removing tissue debris at the site of injury and secreting growth factors or pro-angiogenic factors. Moreover, neutrophils contribute to post-injury tissue repair through dual mechanisms: phagocytosing necrotic debris at the injury site and secreting growth factors (e.g., PDGF) and pro-angiogenic cytokines (e.g., VEGF) (23, 24). This functional overlap with platelets in immune defense highlights their collaborative role in trauma-induced inflammation, where both cell types are activated by tissue damage or inflammatory signals. Concurrently, stressful insults—ranging from traumatic injury to systemic inflammation—induce lymphocytopenia, a phenomenon that reflects the body's adaptive immune recalibration and serves as a marker of immune system resilience (25). Thus, the immune defense and inflammatory cascade triggered by high-energy trauma (e.g., tibial plateau fractures) elicits a characteristic hematological response: thrombocytosis, neutrophilia, and lymphocytopenia. This immunological triad mechanistically underlies the utility of PLR (platelet-to-lymphocyte ratio) and SII (systemic immune-inflammation index) as prognostic markers for TPF severity.

Notably, our results also revealed significant differences in serum Na^+^ and K^+^ levels between the two groups (*P* = 0.016 and *P* = 0.001, respectively), suggesting potential associations between electrolyte imbalance and fracture severity. Severe TPFs (Schatzker types V–VI) are typically caused by high-energy trauma, which often leads to extensive soft-tissue damage, intramedullary hemorrhage, and occult blood loss. Such injuries can induce systemic fluid shifts and hemodilution, disrupting extracellular sodium homeostasis. Additionally, the acute stress response to severe trauma activates the renin-angiotensin-aldosterone system, promoting renal sodium retention and further altering serum Na^+^ levels. For K^+^, trauma-induced cell lysis (from bone and soft-tissue injury) releases intracellular K^+^ into the circulation, while concurrent hypoperfusion or renal dysfunction may impair potassium excretion. These electrolyte disturbances, although not specific to TPFs, likely reflect the magnitude of tissue injury and systemic response, complementing the predictive value of PLR and SII in assessing fracture severity.

There are a variety of factors can affect PLR and SII levels. This study tried to exclude the influence of confounding factors. The PLR is an effective predictor of cardiovascular and rheumatic diseases as has been demonstrated in many studies (13, 15). In addition, PLR has been reported to be associated with the prognosis of many severe injuries, dementia, and malignancies in recent years (10, 16, 26). For example, Wang et al. found that patients with a high PLR (≥189) had a higher one-year mortality rate than patients with low PLR (<189) (10), while Mao et al. demonstrated that PLR >193.55 correlated with malnutrition and advanced cancer staging (12). Similarly, SII has been closely associated with osteoporotic fractures: Fang et al. identified SII ≥ 834.89 as a significant risk factor for postmenopausal osteoporotic fractures (17). To isolate the impact of tibial plateau fracture (TPF)-specific trauma, this study excluded patients with preexisting hematological disorders, chronic infections, malignancies, or conditions known to affect PLR/SII (e.g., osteoporosis, rheumatic diseases). This methodological approach ensures that observed biomarker variations directly reflect the immunological response to TPF severity, rather than being confounded by comorbid conditions.

Tibial plateau fractures are usually caused by high-energy injuries and usually involve the lateral plateau, which is prone to serious knee ligament disorders and neurovascular injuries. Of particular clinical significance, bicondylar tibial plateau fractures (Schatzker types V–VI) are often accompanied by severe intramedullary fractures, comminuted fractures and extensive intramedullary hemorrhage (6). This injury pattern is associated with substantial occult blood loss, which can exacerbate systemic inflammatory responses and contribute to the immunological dysregulation observed in severe TPF cases. The complex fracture architecture of Schatzker V–VI fractures not only pose technical challenges for surgical reconstruction but also correlates with a higher burden of soft-tissue injury and subsequent immune activation, underscoring the need for objective biomarkers to quantify injury severity. However, the Schatzker classification, relying on two-dimensional radiographic imaging, has limitations in fully characterizing the complex injury patterns of TPFs, particularly in visualizing soft-tissue damage, occult hemorrhage, and medullary involvement. In this context, PLR and SII offer unique advantages as systemic biomarkers, reflecting the immunoinflammatory response that correlates with fracture severity. It is important to note that severe TPFs are often accompanied by intravascular volume shifts, hemoglobin dilution, and electrolyte derangements due to fluid sequestration, blood loss, and red blood cell redistribution. These hematological and biochemical changes render markers like hemoglobin (HGB) and electrolytes less reliable for assessing fracture severity, as they primarily reflect systemic volume status rather than the intrinsic severity of the osseous and soft-tissue injury. By contrast, PLR and SII integrate both hematological and inflammatory signals, providing a more comprehensive measure of trauma-induced immune dysregulation (27, 28).

This study is not without limitations. First, PLR and SII values were only obtained at the time of admission, lacking longitudinal monitoring of their dynamic changes during the post-admission course. Second, as a single-center retrospective study with a relatively small sample size, our findings require validation in multi-center, large-cohort retrospective analyses. Third, the study did not elaborate on the detailed physiological mechanisms underlying PLR and SII elevation in severe TPF patients, nor did it provide clinical evidence to guide surgical decision-making. Finally, the exclusion of elderly patients with low-energy injuries may introduce selection bias, potentially limiting the generalizability of our results to broader patient populations.

## 5 Conclusions

In summary, tibial plateau fracture (TPF) represents a highly prevalent intraarticular injury, often leading to extensive soft-tissue damage and profound knee joint dysfunction. This study innovatively identifies the platelet-to-lymphocyte ratio (PLR) and systemic immune-inflammation index (SII) as novel serological markers integrating inflammatory and immune responses, demonstrating their utility in predicting TPF severity. Close monitoring of PLR and SII dynamics upon admission, when combined with radiological assessments, enables clinicians to make more objective and evidence-based judgments about fracture severity. Given their robust predictive value and clinical accessibility, PLR and SII warrant further investigation as potential focal points for future research in trauma immunology and orthopedic precision medicine.

## Data Availability

The raw data supporting the conclusions of this article will be made available by the authors, without undue reservation.
